# *Rothia dentocariosa* Endocarditis in an Unsuspecting Host: A Case Report and Literature Review

**DOI:** 10.1155/2019/7464251

**Published:** 2019-01-23

**Authors:** Stacy Willner, Zaid Imam, Ismail Hader

**Affiliations:** Beaumont, 3601 W 13 Mile Road, Royal Oak, MI 48073, USA

## Abstract

*Rothia dentocariosa*, a gram-positive coccobacillus, is a commensal bacterium that is part of the oropharynx and respiratory tract. In the past, it was known to be a cause for periodontal disease, but in recent years, *Rothia dentocariosa* has been found to be the cause of several other infectious entities, of which endocarditis is the most predominant. We present the case of a healthy 62-year-old female who, after undergoing routine dental cleaning two months prior, developed subacute bacterial endocarditis of the mitral valve, with subsequent cerebral septic emboli causing an occipital hemorrhagic cerebrovascular accident, all secondary to *Rothia dentocariosa*.

## 1. Introduction

Endocarditis is defined as inflammation of the endocardium, which consists of the inner lining of the heart and heart valves. Bacterial infection remains the most common etiology and is most commonly caused by staphylococcus, streptococcus, and enterococcus species. While a *Rothia dentocariosa* infection predominantly causes endocarditis, having endocarditis secondary to *Rothia dentocariosa* remains a rare entity, with few cases documented in medical literature. To this day, there remains no clear consensus on an optimal therapeutic regimen.

## 2. Case Report

A 62-year-old female with no significant past medical history presented to the emergency department in November of 2017 with complaints of arthralgias, most notably in her right knee, left shoulder, and bilateral thighs that made it difficult for her to ambulate. She was also admitted due to a headache that was triggered primarily by coughing. Vital signs on admission were as follows: a blood pressure of 202/90 mmHg, a heart rate of 137 bpm, a respiratory rate of 20, and a temperature of 36.6 Celsius. Physical exam revealed Janeway lesions. She was found to have a neutrophilic leukocytosis, with white blood cell count at 20.4 cells/mm^3^ and neutrophils at 17.4 bil/L. Troponin was elevated at 1.85; this was deemed to be noncardiac in nature as the patient's pain was relieved with ibuprofen and her EKG showed no acute findings. ESR and CRP were elevated at 95 mm/hr and 24.8 mg/dL, respectively. A computed tomography of the brain showed a high-density mass in the right occipital lobe, with surrounding vasogenic edema. The patient continued to deny any visual changes or symptoms other than what was discussed above. An ophthalmologist was consulted to perform a dilated fundus exam, which was positive for small intraretinal hemorrhages that were deemed to be secondary to the patient's hypertension and less likely positive for Roth's spots. There was no evidence of disc edema. A brain MRI with and without gadolinium showed multiple small punctate bilateral areas of acute or subacute infarctions indicative of embolic phenomenon. The hemorrhagic area in the right occipital lobe was again identified, with subtle surrounding enhancement; the differential diagnosis consisted of neoplasm, vascular malformation, or embolic infarction with hemorrhagic conversion. A transthoracic 2D echo was without vegetation, so a transesophageal echo was ordered, and vegetation was shown on the posterior leaflet of the mitral valve. Two blood cultures from admission then came back positive for *Rothia dentocariosa*. Infectious disease was confirmed, and the patient's current antibiotics, which consisted of vancomycin and ceftriaxone, were switched to penicillin G on a continuous pump. The patient remained largely asymptomatic during her admission and was deemed to be stable for discharge from the hospital after a nine-day stay with penicillin G via a continuous pump for a total of six weeks and was planned for a follow-up MRI in three weeks. The repeat MRI came back showing new subacute strokes. The patient was reported, again, to be asymptomatic but was directed to come straight to the emergency department. A repeat transesophageal echo was done and showed the known vegetation on the mitral valve with new vegetation seen on the PICC line and an abscess between the mitral and aortic valves extending into the ascending aorta. The patient then requested transfer to another institution for further evaluation. A repeat transesophageal echo was completed at this outside institution which showed small anterior and posterior mitral leaflet vegetation with no significant destruction and no abscess. A cardiac MRI was then performed which showed a focal delayed enhancement in the apical inferior and lateral wall, likely secondary to coronary arterial embolization. The patient went on to complete the full six weeks of penicillin therapy, remained asymptomatic, and refused a mitral valve replacement. Her follow-up was continued in the cardiology clinic.

## 3. Discussion

Georg and Brown first created the genus “*Rothia*” in 1967; it was to include members of the family Actinomycetaceae, which resemble both *Nocardia* and *Actinomyces* but differ in the physiology and cell wall constituents. The first reported case of endocarditis secondary to *Rothia dentocariosa* was reported in 1978. Prior to that, *Rothia dentocariosa* had a well-established role in the oral flora, with only two more well known cases of infectivity: the first being the case of a periappendiceal abscess in a 19-year-old female in 1975 and the second being the case of a pilonidal cyst in a 17-year-old female in early 1978 [1].

To this day, *Rothia dentocariosa* is mostly recognized as a normal oral pathogen, with low pathogenicity. Infective endocarditis remains the most common manifestation of a *Rothia dentocariosa* infection. In 2002, Boudewijns et al. performed a review of all 20 cases of infective endocarditis secondary to *Rothia dentocariosa*. Of the 20 total cases, 18 had cardiac abnormalities, which predisposed them to endocarditis. The 2 cases without cardiac abnormalities had poor dental hygiene and/or extensive dental procedures, predisposing them to *Rothia dentocariosa* infections. 16 of the 20 patients were cured, with only 6 patients without any reported complications [2].

In 2011, another literature review, along with a case report, was published by Shakoor et al. It cited only two new cases of endocarditis due to *Rothia dentocariosa* since the first literature review in 2002, along with five cases of bacteremia without endocarditis and two cases of sepsis. One of the newly published endocarditis cases was written in Spanish and therefore unavailable, while the other case was about a 55-year-old male with baseline mitral valve prolapse and regurgitation who had undergone a dental extraction prior to the onset of his endocarditis. The high rate of complications reported in the first literature review was mentioned, even though the mortality rate for *Rothia dentocariosa* endocarditis had since remained low [3].

Since the abovementioned literature review in 2007, four new cases of infections due to *Rothia* spp. are found on a routine search in PubMed: one case of *Rothia dentocariosa*-associated peritonitis [4] and three cases of endocarditis, in which two were secondary to *Rothia dentocariosa* [5, 6] and one was secondary to *Rothia aeria* [7]. Of the two cases of endocarditis secondary to *Rothia dentocariosa*, one involved a 34-year-old male, an IV methamphetamine abuser [5], and the other a 58-year-old relatively healthy male [6]. In the case of the IV drug abuser, he originally presented with altered mental status, fever, tachycardia, hypertension, a new diastolic murmur, and new left-sided hemiparesis. On computed tomography of the brain, the patient was found to have new ischemic changes in the right middle cerebral artery territory. A transesophageal echo was then performed, in which large vegetation was seen on the aortic valve. Valve tissue was sent out for culture and came back positive for *Rothia dentocariosa*. The patient was treated for two weeks with ceftriaxone after valve replacement and had complete neurologic resolution [5]. The second case involves a patient who was diagnosed with presumptive endocarditis secondary to a new murmur and nonspecific prodromal symptoms by his PCP. Transesophageal echo revealed moderate-to-severe mitral regurgitation with a thickened and myxomatous mitral valve and a posterior flail leaflet, with no vegetation. The presence of *Rothia dentocariosa* in blood cultures was then confirmed, and the patient was sent home with penicillin G via a continuous pump. Two weeks after discharge, the patient woke up with complete loss of vision in his right eye. After evaluation, he was diagnosed with endogenous endophthalmitis, also secondary to *Rothia dentocariosa*. After full treatment, the patient returned to baseline with no residual deficits [6].

Complications secondary to *Rothia dentocariosa* are common and include, but are not limited to, cerebellar hemorrhages [8], cerebral embolism [9], intracranial hemorrhages [10], vertebral osteomyelitis [11], aortic root abscess [12], abdominal aneurysm [13], perivalvular abscess [14], and brain abscess [15].

The current treatment for *Rothia dentocariosa* endocarditis consists of a 4-6-week regimen of penicillin, with or without gentamicin, or monotherapy with ceftriaxone [5]. In 2014, Ramanan et al. published “Rothia Bacteremia: a 10-Year Experience at Mayo Clinic, Rochester, Minnesota.” 67 patients from 2002 to 2012 with blood cultures positive for *Rothia* spp. were studied and analyzed. Antimicrobial susceptibility was performed on 21% of the isolates; all were susceptible to vancomycin and most were susceptible to beta-lactams; however, 4 were resistant to oxacillin. In the end, it was determined that more data is needed to develop a standard of care for treating *Rothia* spp. [16].

In conclusion, the presented case was a healthy 62-year-old female, who, despite any known risk factors, developed endocarditis secondary to *Rothia dentocariosa*. She sustained numerous subacute strokes secondary to cerebral embolization, while remaining largely asymptomatic. She was successfully treated with penicillin G via a continuous pump for a total of weeks, with subsequent imaging showing almost complete recovery of the mitral valve. This case further proves that *Rothia dentocariosa* can cause endocarditis and should always be a consideration, even in the most unlikely hosts.

## References

[1] Pape J., Singer C., Kiehn T. E., Lee B. J., Armstrong D. (1979). Infective endocarditis caused by *Rothia dentocariosa*. *Annals of Internal Medicine*.

[2] Boudewijns M., Magerman K., Verhaegen J. (2003). *Rothia dentocariosa*, endocarditis and mycotic aneurysms: case report and review of the literature. *Clinical Microbiology and Infection*.

[3] Shakoor S., Fasih N., Jabeen K., Jamil B. (2011). *Rothia dentocariosa* endocarditis with mitral valve prolapse: case report and brief review. *Infection*.

[4] Keng T. C., Ng K. P., Tan L. P., Chong Y. B., Wong C. M., Lim S. K. (2012). *Rothia dentocariosa* repeat and relapsing peritoneal dialysis-related peritonitis: a case report and literature review. *Renal Failure*.

[5] Chowdhary M., Farooqi B., Ponce-terashima R. (2015). *Rothia dentocariosa*: a rare cause of left-sided endocarditis in an intravenous drug user. *The American Journal of the Medical Sciences*.

[6] Fridman D., Chaudhry A., Makaryus J., Black K., Makaryus A. N. (2016). *Rothia dentocariosa* endocarditis: an especially rare case in a previously healthy man. *Texas Heart Institute Journal*.

[7] Collarino R., Vergeylen U., Emeraud C. (2016). Mitral endocarditis due to *Rothia aeria* with cerebral haemorrhage and femoral mycotic aneurysms, first French description. *New Microbes and New Infections*.

[8] Sadhu A., Loewenstein R., Klotz S. A. (2005). *Rothia dentocariosa* endocarditis complicated by multiple cerebellar hemorrhages. *Diagnostic Microbiology and Infectious Disease*.

[9] Almuzara M. N., Mariñansky A. L., Valenzuela V. C., Vay C. A. (2004). Endocarditis due to *Rothia dentocariosa* complicated by septic cerebral embolism. *Enfermedades Infecciosas y Microbiología Clínica*.

[10] Ricaurte J. C., Klein O., Labombardi V., Martinez V., Serpe A., Joy M. (2001). *Rothia dentocariosa* endocarditis complicated by multiple intracranial hemorrhages. *Southern Medical Journal*.

[11] Llopis F., Carratalà J. (2000). Vertebral osteomyelitis complicating *Rothia dentocariosa* endocarditis. *European Journal of Clinical Microbiology & Infectious Diseases*.

[12] Ferraz V., Mccarthy K., Smith D., Koornhof H. J. (1998). *Rothia dentocariosa* endocarditis and aortic root abscess. *The Journal of Infection*.

[13] Weersink A. J. L., Rozenberg-Arska M., Westerhof P. W., Verhoef J. (1994). Rothia dentocariosa endocarditis complicated by an abdominal aneurysm. *Clinical Infectious Diseases*.

[14] Sudduth E. J., Rozich J. D., Farrar W. E. (1993). Rothia dentocariosa endocarditis complicated by perivalvular abscess. *Clinical Infectious Diseases*.

[15] Isaacson J. H., Grenko R. T. (1988). *Rothia dentocariosa* endocarditis complicated by brain abscess. *The American Journal of Medicine*.

[16] Ramanan P., Barreto J. N., Osmon D. R., Tosh P. K. (2014). *Rothia* bacteremia: a 10-year experience at Mayo Clinic, Rochester, Minnesota. *Journal of Clinical Microbiology*.

